# Clinical evaluation of left ventricular function and morphology using an accelerated k-t sensitivity encoding method in cardiovascular magnetic resonance

**DOI:** 10.1186/s13244-019-0750-6

**Published:** 2019-06-13

**Authors:** Antonildes Nascimento Assuncao-Jr, Roberto Nery Dantas-Jr, Renata Margarida do Val, Priscilla Gianotto, Angela dos Santos Marin, Mark Golden, Marco Antonio Gutierrez, Jose Rodrigues Parga, Cesar Higa Nomura

**Affiliations:** 1 Heart Institute (InCor), University of Sao Paulo Medical School, Sao Paulo, Brazil; 2 Canon Medical Systems do Brasil, Sao Paulo, Brazil; 3 Canon Medical Systems Corporation, Otawara, Japan

**Keywords:** Cardiac function test, Cine magnetic resonance imaging, Cardiac imaging techniques, Congestive cardiomyopathies, Ventricular ejection fraction

## Abstract

**Objectives:**

To provide clinical validation of a recent 2D SENSE-based accelerated cardiovascular magnetic resonance (CMR) sequence (accelerated k-t SENSE), investigating whether this technique accurately quantifies left ventricle (LV) volumes, function, and mass as compared to 2D cine steady-state free precession (2D-SSFP).

**Methods:**

Healthy volunteers (*n* = 16) and consecutive heart failure patients (*n* = 26) were scanned using a 1.5 T MRI system. Two LV short axis (SA) stacks were acquired: (1) accelerated k-t SENSE (5–6 breath-holds; temporal/spatial resolution: 37 ms/1.82 × 1.87 mm; acceleration factor = 4) and (2) standard 2D-SSFP (10–12 breath-holds; temporal/spatial resolution: 49 ms/1.67 × 1.87 mm, parallel imaging). Ascending aorta phase-contrast was performed on all volunteers as a reference to compare LV stroke volumes (LVSV) and validate the sequences. An image quality score for SA images was used, with lower scores indicating better quality (from 0 to 18).

**Results:**

There was a high agreement between accelerated k-t SENSE and 2D-SSFP for LV measurements: bias (limits of agreement) of 2.4% (− 5.4% to 10.1%), 6.9 mL/m^2^ (− 4.7 to 18.6 mL/m^2^), − 1.5 (− 8.3 to 5.2 mL/m^2^), and − 0.2 g/m^2^ (− 11.9 to 12.3 g/m^2^) for LV ejection fraction, end-diastolic volume index, end-systolic volume index, and mass index, respectively. LVSV by accelerated k-t SENSE presented good agreement with aortic flow. Interobserver and intraobserver variabilities for all LV parameters were also high.

**Conclusion:**

The accelerated k-t SENSE CMR sequence is clinically feasible and accurately quantifies LV volumes, function, and mass, with short acquisition time and good image quality.

## Key points

- CMR is considered the non-invasive gold standard method for ventricular functional measurements, accomplished mainly through 2D-SSFP cine images
- Accelerated imaging may favor patients with impaired breath-hold or frequent arrhythmia
- Recently developed accelerated CMR cine sequences speed up image acquisition either in temporal, spatial (k-space), or even both domains simultaneously (accelerated k-t SENSE)
- The accelerated k-t SENSE CMR sequence is clinically feasible and accurate for ventricular functional assessment, with short acquisition time and good image quality

## Introduction

Cardiovascular magnetic resonance (CMR) imaging is the non-invasive gold standard modality for quantification of left ventricle (LV) function, volumes, and mass [1]. For these purposes, a 2D cine steady-state free precession (2D-SSFP)-based sequence has been widely used in clinical practice, with high reproducibility and accuracy [1–3]. However, 2D-SSFP requires multiple breath-holds, resulting in prolonged exam duration, and representing a challenge for patients with heart failure symptoms. In addition to that, slice misregistration can be a result of inconsistent breath holding, hampering volumetric estimation [4–6]. Thus, the chosen imaging sequence for determined populations is crucial for delivering fast and accurate diagnostic information. In response to these issues, there have been considerable attempts to accelerate cine sequences, either in temporal, spatial (k-space), or even both domains simultaneously, without considerably reducing spatial or temporal resolutions [7–9].

Accelerated CMR sequences, such as k-t BLAST (broad-use linear acquisition speed-up technique; single receiver coil) and k-t SENSE (sensitivity encoding; multiple receiver coils) consider spatiotemporal correlations throughout the image and rely on undersampling and signal overlap (aliasing) recovery through computational algorithms [7, 8]. Signal correlations and coil sensitivity estimates are obtained in an initial low-resolution acquisition (training stage), and this information is used afterwards for image reconstruction, allowing acceleration by sparsely sampling k-space over time in the main acquisition stage [9].

Recently, a new accelerated 2D k-t SENSE-based cine sequence (accelerated k-t SENSE) was developed to accelerate image acquisition, without performing the training stage [10]. Signal correlations and coil sensitivity estimates are extracted from the acquisition stage data itself during post processing, allowing faster image acquisition [10]. Our goal in the present study is to clinically investigate whether this newly developed accelerated k-t SENSE sequence accurately quantifies LV volumes, function, and mass as compared to the 2D-SSFP cine with acceptable image quality.

## Materials and methods

### Subjects

Healthy volunteers (*n* = 16) and consecutive heart failure patients (*n* = 26) were prospectively enrolled in this study. Exclusion criteria were age under 18 years old, contraindications to CMR (e.g., CMR-incompatible devices, metallic bodies in the eye, intracranial metal clips), irregular heart rhythms, severely impaired breath-hold capacity, claustrophobia, and pregnancy. All patients were clinically referred to CMR assessment of LV volumes, function, and mass. Indications for CMR in patients included non-ischemic (*n* = 20) and ischemic cardiomyopathies (*n* = 6). This study was approved by the Institutional Ethics Committee and all subjects provided written informed consent.

### Accelerated k-t SENSE technique

The rationale and technical details of the acceleration sequence have been described elsewhere [10]. Briefly, accelerated k-t SENSE consists of a k-t SENSE-based SSFP cine sequence that uses no training stage (a previous additional low resolution scan used only for obtaining reference signals) for defining spatiotemporal signal correlations and k-t coil sensitivity maps, thus allowing several fold acceleration. These signal correlations and sensitivity maps are estimated from the acquired image itself during post-processing. As a result, image acquisition is accelerated through undersampling of k-space over time in the main acquisition stage, which provides all correlations needed for final image reconstruction. Therefore, acceleration occurs in both spatial and temporal directions, with partially sampled data, allowing optimization of scan timing.

### MRI protocol

All subjects were prospectively scanned using a 1.5T MRI system (Canon Vantage Titan, Canon Medical System Corporation, Japan). In addition to the standard protocol regarding the clinical indication, two short axis (SA) stacks fully covering both ventricles were acquired and their duration was measured: prospective ECG-triggered accelerated k-t SENSE cine (two slices/breath-hold), and a standard 2D-SSFP cine with parallel imaging and retrospective ECG triggering (one slice/breath-hold).

In order to validate the left ventricle stroke volume (LVSV) obtained by accelerated k-t SENSE, a retrospective phase-contrast flow measurement in the ascending aorta (immediately above sinotubular junction) was performed as a reference in all healthy subjects (VENC 200 cm/s, matrix 256 × 88, temporal/spatial resolution 46 ms/1.48 × 4.31 mm). All cine sequences included standard shimming (64 × 64 mm matrix, voxel size 6.25 × 6.25 mm) and use of view sharing. Imaging parameters are described in Table 1.

**Table 1:** Imaging parameters

	2D-SSFP	Accelerated k-t SENSE
ECG triggering	Retrospective	Prospective
TE/TR (ms)	1.7/3.4	1.7/3.4
FOV (mm)	320 × 360	320 × 360
Image matrix	192 × 192	176 × 192
Spatial resolution (mm)	1.67 × 1.87	1.82 × 1.87
Temporal resolution (ms)	49	37
Slice thickness/spacing (mm)	10/0	10/0
Flip angle (°)	60	60
Bandwidth (Hz/pixel)	977	1302
Cardiac phases (*n*)	25	32
View sharing (segments)	14	11
Breath-holds (*n*)	10–12	5–6
Slices per breath-hold	1	2
Acceleration factor	Parallel imaging^a^	4x
Acquisition duration (s)	161 ± 25	67 ± 14

*TE* echo time, *TR* repetition time, *FOV* field of view, *2D-SSFP* 2D steady-state free precession

^a^Parallel imaging intrinsic acceleration

### Assessment of LV function and morphology

First, all images were de-identified and digitally stored. Then, for quantitative measurements, two experienced cardiovascular imaging specialists (> 5 years of training) analyzed the SA stacks—twice by one reader (time interval between readings of 1 week), using a commercially available software Medis Suite 3.0 (Medis, Leiden, The Netherlands).

All endocardial and epicardial contours were manually drawn as recommended by the Society for Cardiovascular Magnetic Resonance Guidelines [11], covering LV from basal to apical slices, including both papillary muscles and trabeculations as part of the LV cavity. For basal slices, contour was carefully drawn to include the LV outflow tract to the level of the aortic valve cusps, and left atrium was recognized when less than 50% of the blood volume was surrounded by ventricular myocardium [11].

LV volumes (end-diastolic—LVEDV and end-systolic—LVESV), ejection fraction (LVEF), and mass, and their respective indexes corrected for the body surface area, were calculated using the Simpson method. End-diastole phases were chosen as those with the maximum volume in a mid-ventricular slice to provide more consistent estimations of the LV volumes [12]. Quantitative wall motion per-segment analysis (American Heart Association—16-segment model) was also performed, using a centerline method applied to the endocardial and epicardial contours at end-diastole and end-systole [13].

For aortic flow quantification on the healthy volunteers group, the borders of the ascending aorta were traced to include only its cavity. For optimal results, image plane was properly centered and aligned. Aliasing was double checked and, if occurred, velocity encoding sensitivity was set accordingly. The final aortic flow included the estimated coronary arteries flow (mL/beat), calculated as 0.8 × LV mass (g) / heart rate (beats/min) [14].

### Image quality

Quality assessment of cine CMR SA images was performed based on 11 qualitative criteria [15]. This assessment yields a score for LV coverage, presence of artifacts (wrap around, ghosts, metallic and shimming artifacts, image blurring/mis-triggering), signal loss, correct orientation of stack, and adequate gap between slices, assigning individual scores that range from 0 to 18 (the lower the score, the better the image quality) (Table 2).

### Statistical analysis

Continuous variables are shown as mean ± standard deviation, and were compared using paired *t* test or paired Wilcoxon test, as appropriate. Categorical variables were expressed as frequencies (percentages). Agreement of LV parameters on the accelerated k-t SENSE and 2D-SSFP as well as agreement between volumetrically determined LVSV and aortic flow were assessed by Bland-Altman analysis. The same analysis was performed to investigate interobserver and intraobserver agreement. Repeatability coefficients (RC), two times the SDs of the differences between the two measurements, were also calculated for each LV parameter. All statistical analyses were performed using the software R 3.4.3 (The R Foundation for Statistical Computing, Vienna, Austria) and a *p* value < 0.05 was considered statistically significant.

**Table 2:** Image quality of accelerated k-t SENSE and 2D-SSFP method

	2D-SSFP				k-t			
	Score 0	Score 1	Score 2	Score 3	Score 0	Score 1	Score 2	Score 3
Image blurring/mis triggering	35	5	2	0	30	5	5	2
Shimming	40	1	1	0	37	0	2	3
Ghosts	42	0	0	0	36	6	0	0
Correct LV long axes								
LV coverage	42	0	0	0	42	0	0	0
Metallic artifact	42	0	0	0	42	0	0	0
Orientation of stack	42	0	0	0	42	0	0	0
Signal loss	42	0	0	0	42	0	0	0
Slice thickness/Gap	42	0	0	0	42	0	0	0
Wrap around	42	0	0	0	42	0	0	0
Total score^a^	0.33 ± 0.64				0.98 ± 1.25			

*2D-SSFP* 2D steady-state free precession, *LV* left ventricle

^a^mean ± SD

## Results

### Study population, acquisition duration, and image quality

Characteristics of the study population are displayed in Table 3. Most patients were referred to investigate non-ischemic cardiomyopathies and presented mean LVEF of 52 ± 21% (*p* = 0.05), with larger LV volumes than volunteers (*p* values< 0.05). One patient presented infrequent premature ventricular contractions and all individuals had sinus rhythm. The accelerated k-t SENSE reduced by nearly 60% acquisition duration of short-axis images compared to 2D-SSFP (67 ± 14 s versus 161 ± 25 s, *p* < 0.001), with less breath-holds (Table 1).

**Table 3:** Study population

	Patients (*n* = 26)	Volunteers (*n* = 16)	*p* value
Demographics			
Age, years	53 ± 13	43 ± 14	< 0.001
Male, *n* (%)	17 (65)	10 (63)	0.91
BMI, kg/m^2^	29 ± 6	26 ± 3	0.004
BSA, m^2^	1.91 ± 0.27	1.83 ± 0.24	0.33
HR, bpm	68 ± 17	63 ± 17	0.73
PVC, *n* (%)	1 (4%)	–	–
Cardiovascular risk factors			
Hypertension, *n* (%)	17 (65)	1 (6)	–
Diabetes, *n* (%)	8 (31)	–	–
Hypercholesterolemia, *n* (%)	11 (42)	–	–
Smoking, *n* (%)	10 (39)	–	–
Diagnosis			
HCM, *n* (%)	7 (27)	–	–
Ischemic heart disease, *n* (%)	6 (24)	–	–
Chagas heart disease, *n* (%)	3 (11)	–	–
DCM, *n* (%)	3 (11)	–	–
Other cardiomyopathies, *n* (%)	7 (27)	–	–
CMR findings^a^			
LVEDVI, mL/m^2^	92 ± 50	66 ± 10	0.01
LVESVI, mL/m^2^	52 ± 53	26 ± 10	0.04
LVMI, g/m^2^	80 ± 28	54 ± 10	< 0.001
LVSV, mL	76 ± 25	75 ± 15	0.90
LVEF, %	52 ± 21	62 ± 5	0.05

*BMI* body index mass, *BSA* body surface area, *HR* heart rate, *PVC* premature ventricular contraction, *HCM* hypertrophic cardiomyopathy, *DCM* dilated cardiomyopathy, *CMR* cardiovascular magnetic resonance, *LVEDVI* left ventricle end-diastolic volume index, *LVESVI* left ventricle end-systolic volume index, *LVMI* left ventricle mass index, *LVSV* left ventricle stroke volume, *LVEF* left ventricle ejection fraction

Plus-minus values are means ± SD

^a^LV measurements obtained from 2D-SSFP cine

**Fig. 1:**
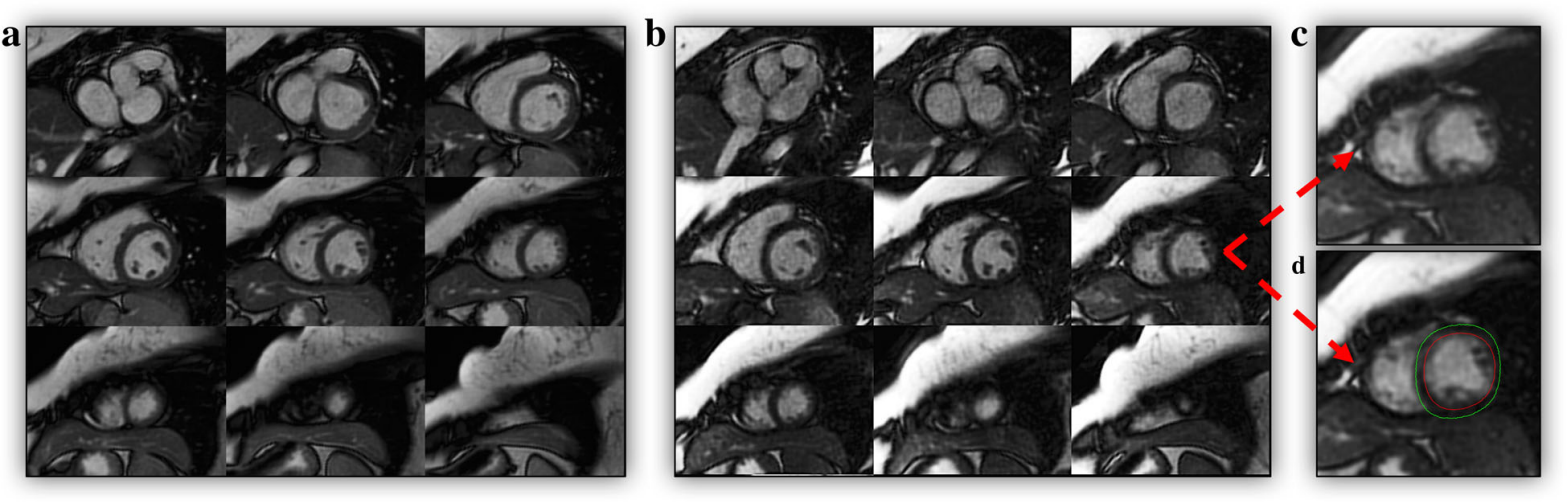
Short-axis images acquired using 2D-SSDFP (**a**) and accelerated k-t SENSE sequence (**b**). Presence of blurring/mis-triggering (**c**) in LV apical slices of accelerated k-t SENSE cine and endocardial/epicardial contours manually corrected (**d**)

Mean quality score resulted in high image quality for both methods, yet 2D-SSFP performed slightly better (0.33 ± 0.64 versus 0.98 ± 1.25, *p* < 0.001) (Table 2). Five accelerated k-t SENSE acquisitions (12%) had score ≥ 3 due to either image blurring/mis-triggering or shimming artifacts (Table 2), but they did not preclude adequate recognition of endocardial/epicardial borders (Fig. 1).

### Agreement between accelerated k-t SENSE and 2D-SSFP cine

**Fig. 2:**
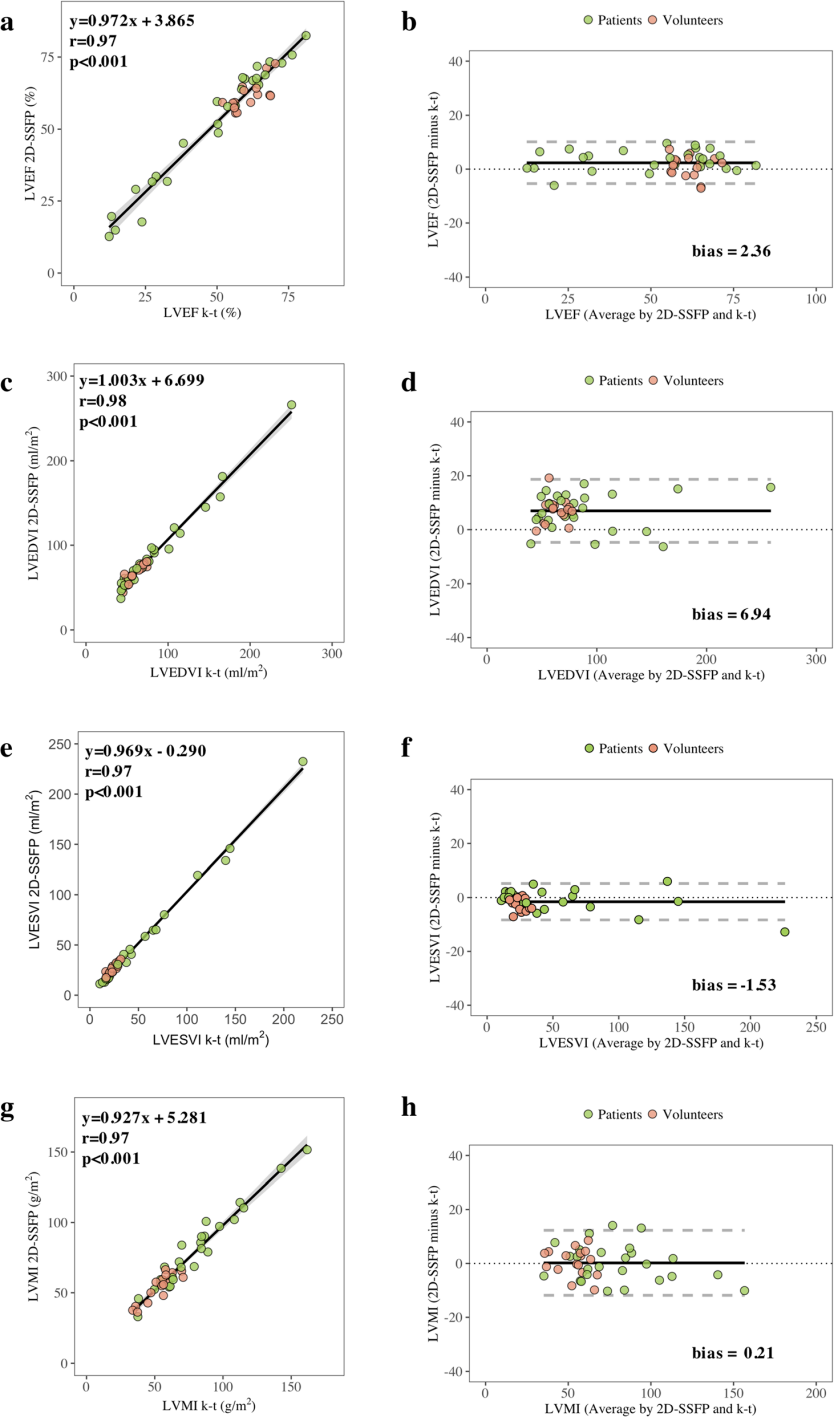
LV volumes, function and mass by accelerated k-t SENSE and 2D-SSFP images. Correlations (**a**, **c**, **e**, **g**) and Bland-Altman analysis (**b**, **d**, **f**, **h**). *LVEF* left ventricular ejection fraction, *LVEDVI* left ventricular end-diastolic volume index, *LVESVI* left ventricular end-systolic volume index, *LVMI* left ventricular mass index

**Fig. 3:**
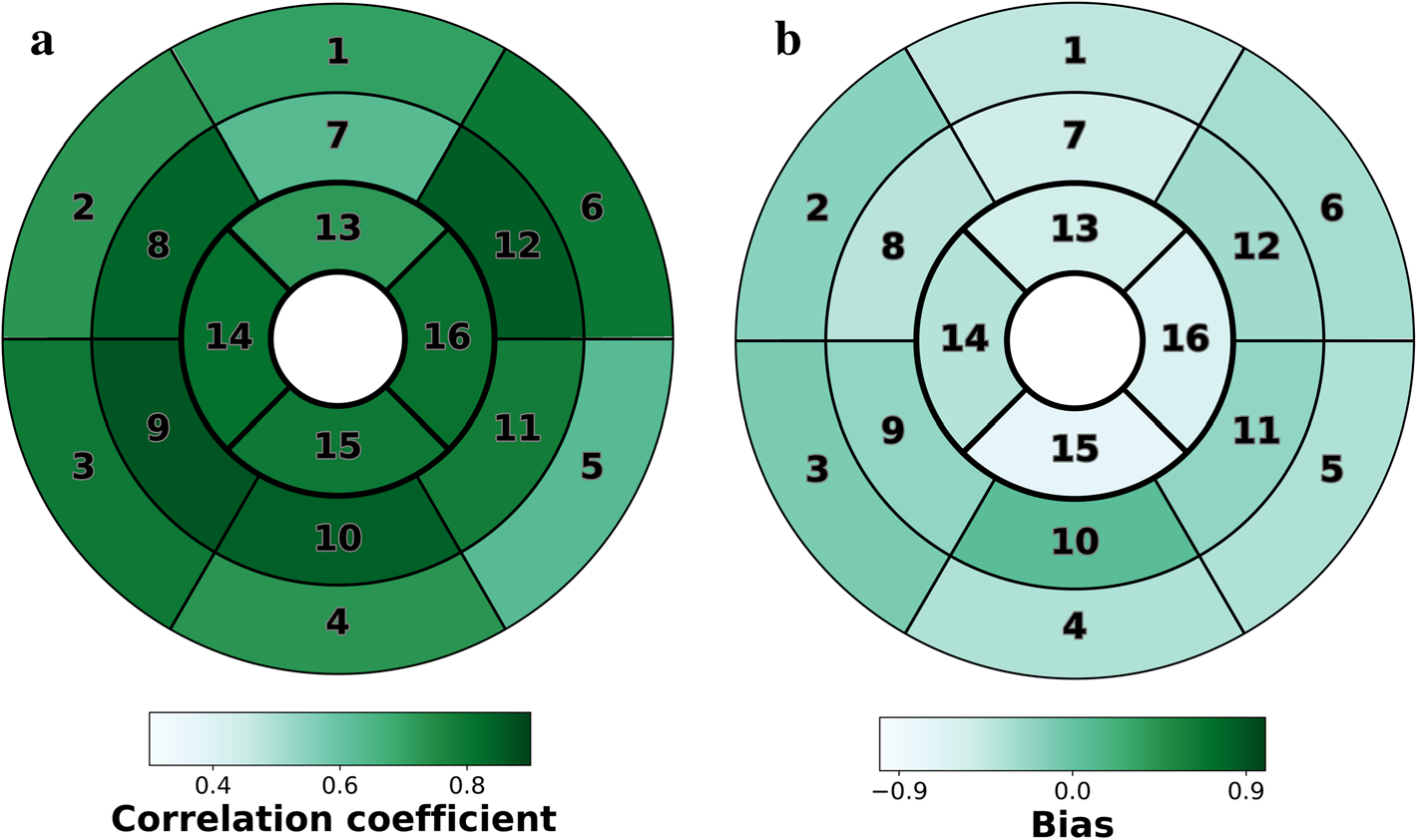
Quantitative regional wall motion correlation and agreement between accelerated k-t SENSE and 2D-SSFP images. 16-Segment American Heart Association bullseye plots indicate the correlation coefficients (**a**) and mean differences or bias (**b**) (in mm) for wall motion assessed by accelerated k-t SENSE and 2D-SSFP cine images

There was a strong correlation between accelerated k-t SENSE and 2D-SSFP for quantification of LV measurements, with high agreement. Mean difference or bias (limits of agreement, LOA) were 2.4% (− 5.4% to 10.1%), 6.9 mL/m^2^ (− 4.7 to 18.6 mL/m^2^), − 1.5 (− 8.3 to 5.2 mL/m^2^), and − 0.2 g/m^2^ (LOA − 11.9 to 12.3 g/m^2^) for LVEF, left ventricle end-diastolic volume index (LVEDVI), left ventricle end-systolic volume index (LVESVI), and left ventricle mass index (LVMI), respectively (Fig. 2). Regional myocardial wall motion analysis also showed good correlation (0.63 ≤ correlation coefficient ≤ 0.87, all *p* values < 0.001) and agreement (− 0.74 ≤ bias ≤ 0.06) between accelerated k-t SENSE and 2D-SSFP (Fig. 3). Interobserver and intraobserver variabilities of accelerated k-t SENSE ranged from 0.1 mL/m^2^ to − 5.2 g/m^2^ and from 0.6 mL/m^2^ to − 4.5 g/m^2^, respectively for LVEDVI and LVMI. RC ranged from 6% (LVEF) to 14 g/m^2^ (LVMI) (Table 4).

**Table 4:** Interobserver and intraobserver reproducibility of accelerated k-t SENSE

	Interobserver			Intraobserver		
	Agreement bias (LOA)	Correlation *r*, *p* value	RC	Agreement bias (LOA)	Correlation *r*, *p* value	RC
LVEF, %	− 2.6 (− 8.2 to 3.6)	0.98, *p* < 0.001	6	1.7 (− 6.5 to 9.8)	0.96, *p* < 0.001	8
LVEDVI, mL/m^2^	0.1 (− 12.2 to 12.2)	0.98, *p* < 0.001	12	0.6 (− 10.8 to 12.1)	0.95, *p* < 0.001	12
LVESVI, mL/m^2^	2.2 (− 7.7 to 9.9)	0.98, *p* < 0.001	10	0.7 (− 9.9 to 8.5)	0.98, *p* < 0.001	9
LVMI, g/m^2^	− 5.2 (− 19.3 to 8.9)	0.95, *p* < 0.001	14	− 4.5 (− 16.5 to 7.5)	0.98, *p* < 0.001	12

*LOA* limits of agreement, *RC* repeatability coefficient, *LVEF* left ventricle ejection fraction, *LVEDVI* left ventricle end-diastolic volume index, *LVESVI* left ventricle end-systolic volume index, *LVMI* left ventricle mass index

### Validation of accelerated k-t SENSE against aortic flow

**Fig. 4:**
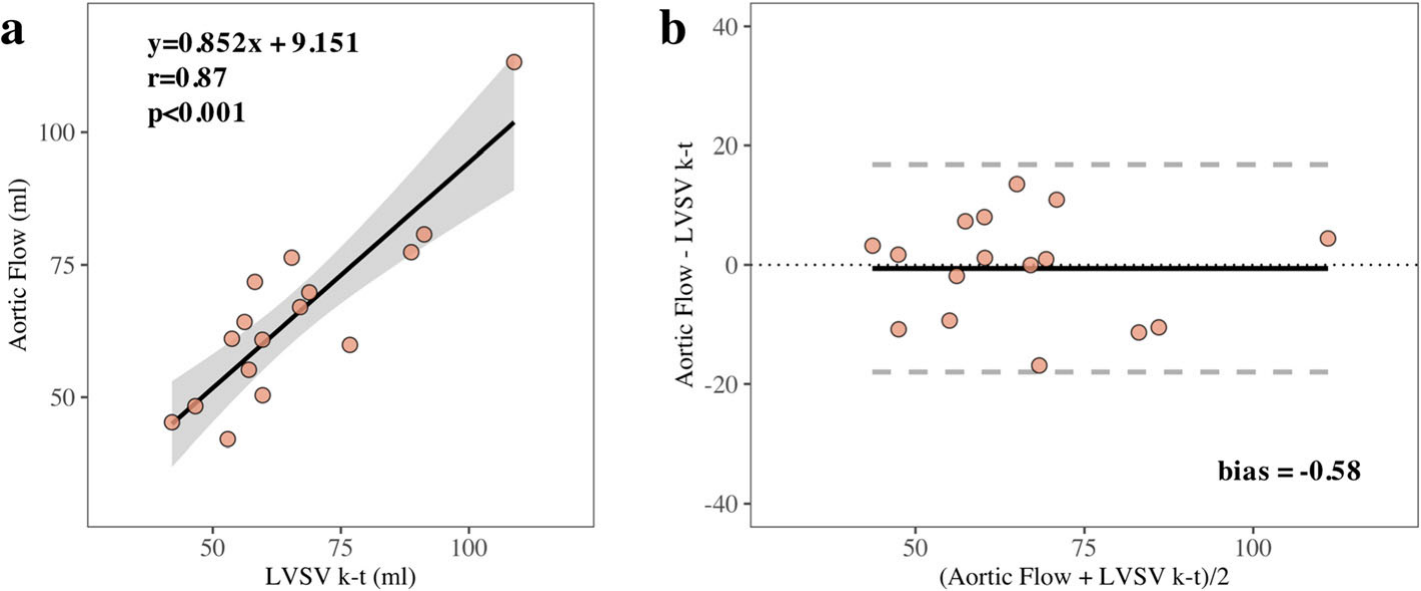
Validation analysis for quantification of stroke volume by accelerated k-t SENSE versus aortic forward flow. Correlation (**a**) and Bland-Altman analysis (**b**). *LVSV* left ventricular stroke volume

The quantification of LVSV by the accelerated k-t SENSE showed a strong correlation and good agreement with aortic flow, slightly underestimating LVSV by 0.58 mL (Fig. 4).

## Discussion

In this study, the accelerated k-t SENSE cine (no training stage approach) was compared to 2D-SSFP for the quantification of LV measurements, with high agreement. The accelerated sequence required half the breath-holds (5 to 6 vs 10 to 12) to cover the entire LV (reducing acquisition time by 60%), and yielded an excellent image quality in 88% of subjects, with accurate assessment of LV function, volumes, and mass as compared to the 2D-SSFP cine.

Agreement between accelerated and standard cines was high for LVEF, with only a slightly wider variability (− 4.1% to 4.3%) [3, 16]. Additionally, accelerated k-t SENSE was quite robust regarding reproducibility for LVEF (RC 6% and 8% for inter- and intraobserver, respectively), closely matching the interobserver reproducibility of 2D-SSFP cine (6% and 12%) [17, 18]. Accelerated k-t SENSE images also provided comparable results in the quantitative regional myocardial wall motion analysis compared to 2D-SSFP.

Likewise, a relevant agreement between the techniques was achieved for LV volumes and mass. Accelerated k-t SENSE promoted a small myocardial mass overestimation and LVEDVI underestimation in a similar fashion of what was found when studying agreement of a 3D k-t BLAST technique with 2D-SSFP cines (despite differences between BLAST and SENSE based sequences) [8]. Other studies regarding spatiotemporal acceleration techniques found similar results, either with compressed sensing or SENSE [19, 20]. Accelerated k-t SENSE had also high accuracy for calculating LVSV using as reference the aortic flow, with a very small overestimation of 0.58 mL. This result is in line with the reported overestimation of LVSV by accelerated sequences in aortic valves [20, 21].

Although the vast majority of patients had excellent image quality, accelerated k-t SENSE yielded, on average, a higher image quality score when compared to the 2D SSFP cine. Indeed, accelerated sequences may favor blurring or residual aliasing artifacts, degrading image quality [22, 23]. However, even in patients with artifacts, images were reasonable for adequate recognition of myocardial borders.

It is remarkable that, despite not reducing acquisition time comparable to neither compressed sensing [20] nor whole heart 3D cine (about 80%) [23], accelerated k-t SENSE reached 60% faster cine acquisition. However, these two other techniques present high computational burden for image reconstruction (precluding immediate assessment of quality or planning next steps) and reporting time (to up 30 min), making accelerated k-t SENSE advantageous.

Some further limitations must be outlined. Firstly, although we included heart failure patients, they had no limiting symptoms and/or significant arrhythmias given the study design. Therefore, additional studies are planned to confirm whether this expressive acquisition time reduction also benefits subjects with more limited breath-hold capabilities. Secondly, despite the possibility of using a wider range of acceleration factors by the accelerated k-t SENSE, faster acquisition (> 4×) accuracy still needs evaluation regarding reliability, as higher acceleration factors with k-t SENSE produced poorer image quality and agreement in previous experience [9]. Finally, retrospective accelerated k-t SENSE was not fully developed by the completion of this work, and may represent further achievements in function assessment.

In conclusion, the accelerated k-t SENSE is feasible and can accurately quantify LV volumes, function, and mass, with good image quality and considerable shortening of acquisition time.

## Abbreviations

2D-SSFP: 2D cine steady-state free precession
BLAST: Broad-use linear acquisition speed-up technique
BSA: Body surface area
CMR: Cardiovascular magnetic resonance
LV: Left ventricle
LVEDVI: Left ventricle end-diastolic volume index
LVEF: Left ventricle ejection fraction
LVESVI: Left ventricle end-systolic volume index
LVMI: Left ventricle mass index
LVSV: Left ventricle stroke volume
NYHA: New York Heart Association
SA: Short axis
SENSE: Sensitivity encoding
